# Effect of body weight on serum homocysteine level in patients with polycystic ovarian syndrome: A case control study

**Published:** 2016-02

**Authors:** Ali I. Al- Gareeb, Wafaa Salah Abd Al- Amieer, Hayder M. Alkuraishy, Thabat J. Al- Mayahi

**Affiliations:** 1 *Department of Clinical Pharmacology* *and Therapeutics, College of Medicine, Al- Mustansiriya University, Baghdad, Iraq.*; 2 *Department of Obstetrics and Gynecology, Al-Kadhymia Teaching Hospital, Baghdad, Iraq.*; 3 *Department of Obstetrics and Gynecology, Al- Kadhymia Teaching Hospital, Baghdad, Iraq.*

**Keywords:** *Homocysteine*, *Body weight*, *Polycystic ovarian syndrome*

## Abstract

**Background:**

Polycystic ovarian syndrome (PCOS) represent one of the common endocrine disorders which influence around 8% of reproductive women whom usually suffering from obesity and increase cardiovascular risk. Serum homocysteine levels are associated with bad impact on endothelial functions and considered as an independent risk factor for cardiovascular disease.

**Objective:**

The aim was to study the level of plasma homocysteine in obese and non-obese Iraqi patients with PCOS.

**Materials and Methods:**

This study was carried out on 207 women. Of theme, 101 women with PCOS and 106 PCOS- free women served as controls. Blood sample was taken from each participant on the 2^nd^ day of menstruation morning after an overnight fasting. Serum levels of follicle-stimulating hormone (FSH), luteinizing hormone (LH), free testosterone and androstenedione were measured. Moreover, total lipid profile and plasma homocysteine levels were measured in both groups.

**Results:**

Sixty percent of PCOS women were overweight or obese and 56% of them had a waist circumference >88cm. Moreover plasma homocysteine concentrations were found to be higher in patients with PCOS (11.5±5.41μmol/L) as compared with control (8.10±1.89 μmol/L) (p<0.002). Furthermore the homocysteine concentrations were 13.19±5.97 μmol/L and 9.38±2.99 μmol/L in both obese and normal-weight PCOS women respectively which was significantly higher than obese (p<0.002) and normal-weight (p<0.004) control women.

**Conclusion:**

Increase in body weight is not an independent risk factor to increase plasma homocysteine levels in PCOS women.

## Introduction

Hyperandrogenism, ovulatory dysfunction and polycystic ovaries are the main features of polycystic ovary syndrome (PCOS) (1). Clinical manifestations, such as hirsutism, acne and male pattern baldness, mirror the elevated amounts of circling androgen that outcome from over top emission of hormone from ovary and/or adrenal organs. Aggravation of ovarian capacities showed clinically as inconsistency of menstrual cycle and infertility. Ultrasound examination of polycystic ovaries uncovered an expanded number of little antral follicles with ended advancement and a hypertrophied theca cell layer (1-3). Apart from abnormal hair growth, menses irregularity, and infertility, patients with PCOS show wide variety of metabolic abnormalities including adverse lipid profile, insulin resistance associated with hyperinsulinemia, and obesity (4-6). 

Patients with PCOS are highly vulnerable to develop cardiovascular disease. Homocysteine is a non-protein forming, sulfur containing amino acid that plays an important role in methionine and folate metabolism (7). The definition of hyperhomocysteinemia is not well defined in literature; Cutoffs have varied from 8.3-13.75 μmol/L and differ by age and ethnicity (8-11). 

However, hyperhomocysteinemia is commonly defined as concentrations greater than 12.0 μmol/L (12). Since 1969, the basis for hypothesis that elevated blood homocysteine levels may be a risk factor for cardiovascular disease in general population was formed. The mechanisms by which homocysteine induces the development and progression of vascular disease have not been fully elucidated (13). However, current research supports the role for homocysteine as mediator for endothelial damage and dysfunction (14). This would be a key factor in subsequent impaired endothelial dependent vasoreactivity and decreased endothelium thromboresistance. This in tum would prompt vessels in patient with hyperhomocysteinemia to develop atherogenesis.

In recent years homocysteine has been portrayed as an independent risk factor for disease of heart and vascular tree (15). Plasma homocysteine levels have been indicated to correspond with blood pressure, body weight and insulin receptor sensitivity, since hypertension, obesity and hyperinsulinemia are habitually experienced elements of PCOS, it appears to be coherent to guess that raised homocysteine levels could be another element of PCOS and this component may add to expanded extensiveness of cardiovascular disturbances in patient with PCOS (16-19).

This study was designed to evaluate concentrations of plasma homocysteine in obese and non-obese patients with PCOS.

## Materials and methods

This case-control study was conducted in Department of Obstetrics and Gynecology at Al-Kadhemia Teaching Hospital in collaboration with Department of Clinical Pharmacology and Therapeutics at College of Medicine, Al-Mustansiriya University between December 2013 and March 2014 and was approved by scientific committee of Al-Mustansiriya College of Medicine, Baghdad, Iraq. Informed consent was obtained from all patients and all patients were treated.

207 women, aged 18-42 years, were consecutively enrolled the study. 101 patients suffered from PCOS, and the remaining 106 PCOS- free patients served as controls. PCOS was diagnosed according to Rotterdam ESHRE/ASRM-sponsored PCOS Consensus Workshop Group 2003, i.e. if two of following criteria were present: menstrual cycle disorders (oligomenorrhea, defined as cycles lasting longer than 35 days, or amenorrhea, defined as cycles lasting longer than 3 months), clinical or biochemical signs of hyperandro-genism (hirsutism) with a Ferriman- Gallwey score of more than eight or obvious acne or alopecia or an elevated testosterone (normal range 0.5-2.6 nmol/l) and/or androstenedione (normal range 0.3-3.3 ng/ml), and sonographically diagnosed polycystic ovaries (at least one ovary with at least 12 follicles with a diameter of 2-9 mm each or a volume >10 ml) (20).

Exclusion criteria for all subjects including current or previous use of oral contraceptive pills (within 6 months), drugs like metformin, phenytoin, folic acid, vitamins, antiandrogens, antidiabetics, statin, glucocorticoids, cigarette smoking, known case of hypertension, diabetes mellitus and cardiovascular disease. Patients with tubal sterility, male causes of sterility, and patients with recurrent miscarriages but without any endocrine disorders or causes served as controls. Same exclusion criteria as case group were used for control group. Entire women were subjected to thorough physical examination and laboratory tests. Endocrine variables as well as homocysteine levels were determined on the 2^nd^ day of menstruation after an overnight fasting.

During the same visit, all subjects underwent anthropometric measurement. Blood samples were drawn from participants; the blood samples were immediately put on crushed ice. In the following hour samples were centrifuged at 3000 gr for 20 min at 4^o^C and immediately frozen until analysis. The serum levels of FSH and LH in serum were measured by enzyme-linked immunosorbent assay (ELISA). Serum testosterone and androstenedione were measured using an ELISA and commercial kits (IBL, Hamburg). Serum glucose, HDL cholesterol, total cholesterol, and TG were measured using an enzymatic calorimetric method with an autoanalyzer (Mindray BS-Clinical Chemistry Analyzer, Guangzhou Shihai Medical Equipment Co, Guangdong, China). 

Serum homocysteine (total) levels were measured using radioenzymatic assay based on conversion of homocysteine to S-adenosylhomocysteine in presence of adenosine and S-adenosylhomocysteine hydrolase. Dithioerythritol was used as reductant, and radioactive S-adenosyl-homocysteine was quantified by HPLC 21.

**Statistical analysis**

Data were statistically described in terms of range, mean±SD and relative frequencies (%). Comparison between different groups was done using unpaired t-test for comparing continuous data while Chi square (^2^) test was performed for comparing categorical data. A probability value (p-value) less than 0.05 was considered significant. All statistical calculations were done using computer statistical programs SPSS ver. 20 (Statistical Package for the Social Science; SPSS Inc. Chicago, IL, USA).

## Results

The members of this study were 207; of them 101 female with PCOS (PCOS group) while the remaining 106 participant female were free from PCOS (control group). The mean age of participants in study group was 24.86 years (18-39 years) in PCOS and 25.41 years in control group (19-42 years). There was no statistical difference between groups regarding the age (p=0.468). The mean values of BMI and waist circumference were considerably higher in PCOS subjects than in control subjects.

In PCOS group, sixty subjects were suffering from increase their body weight (BMI of ≥25 kg/m^2^) and 41 subjects were found to be with normal body weight whereas in control group, 24 subjects were with increase body weight but the remaining 82 subjects were with BMI of ≤25 kg/m^2^. Regarding waist circumference, 54 (53.4%) women with PCOS were with a circumference of >88 cm compared to 21 (19.6%) women of controls p<0.01.

Hirsutism is one of common clinical features of PCOS. In our study 57 of PCOS women (56.43%) had hirsutism as compared to 13 women of control (12.26%) had hirsutism (p<0.001). Women with PCOS had signiﬁcantly elevated testosterone, androstenedione, and LH levels as well as LH/FSH ratio in comparison with the control group, (p<0.001) (Table I). Moreover the majority of PCOS subjects (66 patients) had polycystic appearing ovaries on pelvic sonography in comparison to only 9 participants of control group (p<0.0001).

Regarding fasting glucose level, it has been found that there was no significant difference in fasting glucose concentrations between both groups (p>0.5) (Table II). Regarding lipid profile in both groups, it has been found that differences between them in mean serum levels of total cholesterol and LDL-cholesterol were statistically non-significant whereas mean triglyceride concentration was significantly higher and HDL-cholesterol concentration was significantly lower in PCOS group (Table II). 

Other than checked contrasts in hormonal parameters, and lipid profiles between PCOS group and controls, the present study showed proof of essentially higher homocysteine levels in PCOS patients as compared with control subjects. The mean homocysteine level in PCOS group was 11.5±5.41 μmol/L and was 8.10±1.89 μmol/L in control group (Table II). 

Hoisted homocysteine was noted in PCOS patients regardless of their body weight as opposed to control group. The mean homocysteine levels in obese and non-obese group with PCOS were 13.19±5.97 μmol/L and 9.38±2.99 μmol/L respectively (Table III). Among the controls, obese subjects had a mean total homocysteine level of 8.62±3.15 μmol/L whereas, non-obese subjects had mean homocysteine level of 7.48±1.53 μmol/L. It is nice to mention that serum homocysteine levels, regardless whether the participant suffer or not from PCOS, were found to be higher in obese group compared to non-obese group, and the difference was statistically significant (Table III). 

Moreover the previous table pointed clearly that elevated plasma homocysteine level occurs in both non-obese and obese PCOS as opposed to non-obese and obese control. It is interesting to state that about 37% of PCOS patients had hyperhomocysteinemia (HCy >12 μmol/L) in comparison to about only 10% of control group (p<0.001) regardless the state of their body weight (Table IV).

**Table I:** Serum hormone levels of women in both of PCOS and control groups

**Variables**	**PCOS**	**Control**	**p-value** *
Testosterone (nmol/l)	3.30 ± 1.9	2.29 ± 1.41	<0.01
Androstenedione (ng/ml)	4.08 ± 2.3	2.89 ± 1.1	<0.01
FSH (IU/L)	3.39 ± 2.03	3.19 ± 1.51	NS
LH (IU/L)	7.93 ± 5.81	3.69 ± 2.8	<0.01
FSH/LH	2.33 ± 0.81	1.15 ± 0.13	<0.01

LH: Luteinizing hormone

FSH: Follicle-stimulating hormone

* Using unpaired t test. Data are presented as mean±SD.

**Table II:** Plasma glucose, serum lipids and plasma homocysteine levels in both of PCOS and control groups

**Variables**	**PCOS**	**Control**	**p-value** *
Fasting glucose (mg/dl)	95.22 ± 7.87	89.21 ± 5.78	NS
Total cholesterol (mg/dl)	174.87 ± 7.62	176.21 ± 8.90	NS
Triglyceride (mg/dl)	134.87 ± 15.09	101.56 ± 12.32	<0.01
HDL-cholesterol (mg/dl)	43.24 ± 10.89	57.56 ± 11.04	<0.01
LDL- cholesterol (mg/dl)	105.32 ± 24.55	99.22 ± 24.87	NS
Homocysteine (μmol/L)	11.5 ± 5.41	8.10 ± 1.89	<0.002

* Using unpaired t test

**Table III:** Homocysteine levels in obese and non-obese subjects in both of PCOS and control groups

**Group**	**Obese (BMI ≥25)**		**Non obese (BMI <25)**		**p-value** *
	**No**	**HCy (mean±SD) μmol/L**	**No**	**HCy (mean±SD) μmol/L**	
PCOS (n=101)	60	13.19 ± 5.97	41	9.38 ± 2.99	0.02
Control (n=106)	24	8.62 ± 3.15	82	7.48 ± 1.53	0.04
p*		0.01		0.01	

* Using unpaired t test

**Table IV:** Homocysteine levels in both of PCOS and control groups

**Group**		**No. (%)**	**BMI (mean±SD)**	**HCy (mean±SD)**
HCy <12 μmol/L				
	Control	90 (89.10)	24.32±2.01	7.11 ± 1.22
	PCOS	66 (65.34)	25.88±2.95	8.51 ± 1.32
	p-value	0.001*	0.06**	0.001**
HCy >12 μmol/L				
	Control	11 (9.90)	29.02±2.11	13.11 ± 1.01
	PCOS	35 (36.66)	28.78±2.23	16.51 ± 3.41
	p-value	0.001*	0.08**	0.02**

* Chi square (^2^) test

** using unpaired t test

## Discussion

The present study showed that serum homocysteine levels were significantly higher in PCOS group than control group, whether they were obese or non-obese. Moreover lipid and lipoproteins abnormalities were more in women with PCOS than that of age matched controls. This study revealed clearly that obesity is signiﬁcantly higher in patients with PCOS than in corresponding control group i.e. about 60% of PCOS vs 20% of control were obese. Moreover PCOS group has been found to have a high prevalence of upper body obesity (android obesity) as demonstrated by increased waist circumference and waist-hip ratio compared to control group. 

These results are in concurrence with earlier studies which demonstrated that increase in body weight is a characteristic finding in women with PCOS, that’s to say that about 40-80% of women with this condition were accounted for to be overweight or obese (22-23). It is surely understood that androgens assume a vital part in determination of body build. Men have more muscle to fat ratio with more remarkable spread of fat in upper part of the body (android) contrasted with women, who have a tendency to amass fat in the lower segment of the body (gynoid). Vague initially reported that predominance of diabetes, hypertension, and atherosclerosis was higher in women with android obesity contrasted with gynoid one (24). 

Incessant experience to higher testosterone levels in women with PCOS may alter fat distribution in their bodies. Support for this speculation is given by observing the clinical and biochemical influence of exogenous androgen that given to normal body weight female to male transsexuals that prompt increments in visceral fat and adversely affect insulin receptors sensitivity (25). Moreover hormonal changes in women after menopause when levels of estrogen decline so there body tissue will exposed to increasing influence of higher androgen leads to increase visceral fat regardless of their body weight (26). Even in animal, early exposure to high dose of androgen shown to alter body fat distribution in favoring of central body fat accumulation (27). So higher level of androgens in patients with PCOS may be claimed to be the cause of their android type of obesity.

It is interesting to mention that in addition to prevalence of central obesity in PCOS group, another important cardiovascular risk factors namely adverse lipid profile is found to be more in comparison to control group. It has been found that PCOS women had significant increased levels of triglycerides whereas HDL-C levels were significantly decreased and these finding of course indicating more risk for cardiovascular disease. This results are in agreement with many previous studies which showed that PCOS were associated with more pronounced atherogenic lipid profile whereas it contradict with other less previous studies that did not revealed any difference in lipid parameters in PCOS women compared with controls (28-31).

The possible explanation for this derangements of lipid profile that observed in patients with PCOS might attributed to increase levels of androgens that associated with development of PCOS and this higher level of androgens result in impairment of insulin receptor sensitivity which leads to metabolic derangement with its atherogenic potential (32, 33). This is strengthened by perception that usual unsettling influence of lipid parameters seen in PCOS is connected with insulin resistance (34). It is well-known that insulin resistance result in accelerated peripheral lipolysis which translated in breakdown of subcutaneous triglycerides in to free fatty acid that increased in serum. This result in increment of input of free fatty acid to liver and this has stimulatory effects on hepatocyte to synthesize more and more VLDL which eventually prompts hypertriglyceridemia (35). 

It has been proposed previously that insulin hinders the declaration of microsomal triglyceride protein, which is in charge of emission of apolipoprotein B (apoB) and VLDL (36). Insulin resistance prompts hepatic overproduction of apoB and VLDL and, eventually, to hypertriglyceridemia (29). Additionally atherogenic adjustments of LDL cholesterol toward more atherogenic particles (more small and dense) have been illustrated (33). 

Moreover androgens, particularly testosterone, may participates through its inhibitory effects on central fat metabolizing lipase enzyme and this may account to central obesity as its mentioned above (37). Moreover high androgens levels previously proven to have stimulatory influence on hepatic lipase that participate in metabolism of HDL particles and this might explain the decrement in levels of HDL observed in patient with PCOS in our study (25). In present study, serum levels of homocysteine in Iraqi women with PCOS interestingly found to be higher than control group (p=0.002) and this finding are in agreement with many previous results whereas it contradict with other previous study that did not determine signiﬁcant differences in homocysteine levels between women with PCOS and control group (38-41). 

It is good to state that in present study it has been found that higher homocysteine levels were detected in women with PCOS regardless of their body weight status. It's difficult to declare weather increase homocysteine levels predispose to PCOS or PCOS result in increment of homocysteine level in serum. The importance of association of increase serum levels of homocysteine in women with PCOS comes from the bad impact of high homocysteine levels on heart and vascular tree. It's well documented that patients with PCOS are more prone to develop cardiovascular complication so changing of homocysteine levels in those patient may have important role in development of the syndrome, or at least its progression map. 

Furthermore certain studies shown that, mean plasma homocysteine levels are significantly higher in Insulin resistant PCOS patients when compared with non-insulin resistant PCOS patients regardless of BMI which indicates relationship of homocysteine with plasma levels of insulin (42, 43). In the present study insulin resistance didn't addressed but there were slightly non-significant increase in fasting blood glucose levels in women with PCOS group when compared to control group. These finding further highlighted important of considering plasma level of homocysteine and its adverse role in metabolic complications of patients with PCOS.

## Conclusion

In conclusion, this study revealed clearly that increase body weight, adverse lipid profile and increase in plasma homocysteine levels are more prevalent in patients with PCOS. Moreover the increment in plasma homocysteine levels is present in both of obese and non-obese patients with PCOS.

## References

[1] Dumitrescu R, Mehedintu C, Briceag I, Purcarea VL, Hudita D (2015). The polycystic ovary syndrome: an update on metabolic and hormonal mechanisms. J Med Life.

[2] Burt Solorzano CM, Beller JP, Abshire MY, Collins JS, McCartney CR, Marshall JC (2012). Neuroendocrine dysfunction in polycystic ovary syndrome. Steroids.

[3] Roland AV, Moenter SM (2014). Reproductive neuroendocrine dysfunction in polycystic ovary syndrome: insight from animal models. Front Neuroendocrinol.

[4] Reaven GM (2011). Insulin resistance: the link between obesity and cardiovascular disease. Med Clin North Am.

[5] González F (2015). Nutrient-Induced Inflammation in Polycystic Ovary Syndrome: Role in the Development of Metabolic Aberration and Ovarian Dysfunction. Semin Reprod Med.

[6] Mukherjee S, Maitra A (2010). Molecular, genetic factors contributing to insulin resistance in polycystic ovary syndrome. Indian J Med Res.

[7] Gurda D, Handschuh L, Kotkowiak W, Jakubowski H (2015). Homocysteine thiolactone and N-homocysteinylated protein induce pro-atherogenic changes in gene expression in human vascular endothelial cells. Amino Acids.

[8] Prajapati J, Jain S, Virpariya K, Rawal J, Joshi H, Sharma K (2014). Novel atherosclerotic risk factors and angiographic profile of young Gujarati patients with acute coronary syndrome. J Assoc Phys Ind.

[9] Cao C, Hu J, Dong Y, Zhan R, Li P, Su H (2015). Gender differences in the risk factors for endothelial dysfunction in Chinese hypertensive patients: homocysteine is an independent risk factor in females. PLoS One.

[10] Papandreou D, Mavromichalis I, Makedou A, Rousso I, Arvanitidou M (2006). Reference range of total serum homocysteine level and dietary indexes in healthy Greek schoolchildren aged 6-15 years. Br J Nutr.

[11] Feng SQ, Ye P, Luo LM, Xiao WK, Xu RY, Wu HM (2012). Relationship between serum homocysteine and metabolic syndrome: A cross-sectional study. Zhonghua Liu Xing Bing Xue Za Zhi.

[12] Kang, SS, Wong PW, Malinow MR (1992). Hyperhomocyst (e) inemia as a risk factor for occlusive vascular disease. Annu Rev Nutr.

[13] Wilcken DE, Wilcken B (1976). The pathogenesis of coronary artery disease. A possible role for methionine metabolism. J Clin Invest.

[14] Van Guldener C, Stehouwer CD (2000). Hyperhomocysteinemia, vascular pathology, and endothelial dysfunction. Semin Thromb Hemost.

[15] Wong YY, Golledge J, Flicker L, McCaul KA, Hankey GJ, Van Bockxmeer FM (2013). Plasma total homocysteine is associated with abdominal aortic aneurysm and aortic diameter in older men. J Vasc Surg.

[16] Sule AA, Chin TJ, Khien LH (2012). Recurrent unprovoked venous thromboembolism in a young female patient with high levels of homocysteine. Int J Angiol.

[17] Chen H, Sun Y, Wang X, Si Q, Yao W, Wan Z (2015). Association of cardiometabolic risk profile with prehypertension accompany hyperhomocysteinaemia. Clin Exp Hypertens.

[18] Esmaeilzadeh S, Andarieh MG, Ghadimi R, Delavar MA (2014). Body mass index and gonadotropin hormones (LH &amp, FSH) associate with clinical symptoms among women with polycystic ovary syndrome. Glob J Health Sci.

[19] Diamanti- Kandarakis E, Dunaif A (2012). Insulin resistance and the polycystic ovary syndrome revisited: an update on mechanisms and implications. Endocrine Rev.

[20] Rotterdam ESHRE/ASRM-Sponsored PCOS consensus workshop group (2004). Revised 2003 consensus on diagnostic criteria and long-term health risks related to polycystic ovary syndrome (PCOS). Hum Reprod.

[21] Refsum H, Hellimd S, Uelalid PM (1985). Radioenzymje determination of homocyateine in plasma and urine. Clin Chem.

[22] Unluer AN, Findik RB, Sevinc N, Karakaya J (2013). Comparison of HbA1c levels in obese and non-obese polycystic ovarian patients. Clin Exp Obstet Gynecol.

[23] Legro RS (2012). Obesity and PCOS: implications for diagnosis and treatment. Semin Reprod Med.

[24] Vague J (1956). The degree of masculine differentiation of obesities: A factor determining predisposition to diabetes, trosclerosis, gout, and uric calculous disease. Am J Clin Nutr.

[25] Elbers JM, Asscheman H, Seidell JC, Megens JA, Gooren LJ (1997). Long-term testosterone administration increases visceral fat in female to male transsexuals. J Clin Endocrinol Metab.

[26] Moran C, Arriaga M, Arechavaleta- Velasco F, Moran S (2015). Adrenal androgen excess and body mass index in polycystic ovary syndrome. J Clin Endocrinol Metab.

[27] Alexanderson C, Eriksson E, Stener- Victorin E, Lystig T, Gabrielsson B, Lönn M, Holmäng A (2007). Postnatal testosterone exposure results in insulin resistance, enlarged mesenteric adipocytes, and an atherogenic lipid profile in adult female rats: comparisons with estradiol and dihydrotestosterone. Endocrinology.

[28] Harrison CL, Stepto NK, Hutchison SK, Teede HJ Teede (2012). The impact of intensified exercise training on insulin resistance and fitness in overweight and obese women with and without polycystic ovary syndrome. Clin Endocrinol.

[29] Li XJ, Yu YX, Liu CQ, Zhang W, Zhang HJ, Yan B et al (2011). Metformin vs thiazolidinediones for treatment of clinical, hormonal and metabolic characteristics of polycystic ovary syndrome: a meta-analysis. Clin Endocrinol.

[30] Valkenburg O, Steegers-Theunissen RP, Smedts HP, Dallinga-Thie GM, Fauser BC, Westerveld EH (2008). A more atherogenic serum lipoprotein profile is present in women with polycystic ovary syndrome: a case-control study. J Clin Endocrinol Metab.

[31] Huang J, Ni R, Chen X, Huang L, Mo Y, Yang D (2010). Metabolic abnormalities in adolescents with polycystic ovary syndrome in south China. Reprod Biol Endocrinol.

[32] Dilbaz B, Ozkaya E, Cinar M, Cakir E, Dilbaz S (2011). Cardiovascular disease risk characteristics of the main polycystic ovary syndrome phenotypes. Endocrine.

[33] Boden G (2011). Obesity, insulin resistance and free fatty acids. Cur Opin EndocrinDiabetes Obes.

[34] Baldani DP, Skrgatić L, Goldstajn MS, Zlopasa G, Oguić SK, Canić T (2012). Clinical and biochemical characteristics of polycystic ovary syndrome in Croatian population. Coll Antropol.

[35] Azziz R, Carmina E, Dewailly D, Diamanti-Kandarakis E, Escobar-Morreale HF, Futterweit W (2006). Positions statement: criteria for defining polycystic ovary syndrome as a predominantly hyperandrogenic syndrome: an Androgen Excess Society guideline. J Clin Endocrinol Metab.

[36] Thornton EC, Von Wald T, Hansen K (2015). Polycystic Ovarian Syndrome: A Primer. SD Med.

[37] Baldani DP, Skrgatic L, Ougouag R (2015). Polycystic Ovary Syndrome: Important Underrecognised Cardiometabolic Risk Factor in Reproductive-Age Women. Int J Endocrinol.

[38] Feng SQ, Ye P, Luo LM, Xiao WK, Bai YY, Feng D (2012). Associations of plasma homocysteine and high-sensitivity C- reactive protein levels with arterial stiffness in Chinese population: a community- based study. Chin Med J (Engl).

[39] Loverro G, Lorusso F, Mei L, Depalo R, Cormio G, Selvaggi L (2002). The plasma homocysteine levels are increased in polycystic ovary syndrome. GynecolObstet Invest.

[40] Wijeyaratne CN, Nirantharakumar K, Balen AH, Barth JH, Sheriff R, Belchetz PE (2004). Plasma homocysteine in polycystic ovary syndrome: does it correlate with insulin resistance and ethnicity. Clin Endocrinol.

[41] Mancini F, Cianciosi A, Reggiani GM, Facchinetti F, Battaglia C, de Aloysio D (2009). Endothelial function and its relation- ship to leptin, homocysteine, and insulin resistance in lean and overweight eumenorrheic women and PCOS patients:a pilot study. Fertil Steril.

[42] Maleedhu P, M V, SS BS, Kodumuri PK, Devi DV (2014). Status of Homocysteine in Polycystic Ovary Syndrome (PCOS). J Clin Diagn Res.

[43] Ilhan T, Berrin C, Zeynep C, Erdem T (2005). The Plasma homocysteine concentrations and relationship with insulin resistance in young women with polycystic ovary syndrome. Turk J Endocrinol Metab.

